# CAREGIVING AND COGNITIVE IMPAIRMENT IN MEXICO AND THE US

**DOI:** 10.1093/geroni/igac059.666

**Published:** 2022-12-20

**Authors:** Phillip Cantu, Sunshine Rote, Joseph Gaugler

## Abstract

Dementia is one of the most common causes of disability and dependence in the world. The growing dementia population in Mexico is exemplary of many low- and middle-income countries (LMICs). While the U.S. has a highly developed formal long-term care system, the use of institutional care among Latinos has been low. Mexico lacks a publicly financed long-term care system, and it does not have a national-level mandatory registry of institutions, compulsory standards of care, nor a regulatory body to oversee management, quality of care standards for services, or the accreditation and evaluation of service providers. There are no policies, public programs, or services to provide dependency care, including support for people living with dementia and their family caregivers. As in other LMIC’s, there is limited public support for the aging population in Mexico, leaving families with the main responsibility of providing care and economic security for older adults. “Informal” and family caregivers for older adults with cognitive impairment are critical components of how older adults with cognitive impairment are able to remain in the community. This symposium will reflect the broad spectrum of caregivers and care context in the U.S. and Mexico to shed light on sources of care for older adults living with cognitive impairment.

